# Pediatric Acute Respiratory Distress Syndrome: Fluid Management in the PICU

**DOI:** 10.3389/fped.2016.00021

**Published:** 2016-03-21

**Authors:** Sarah A. Ingelse, Roelie M. Wösten-van Asperen, Joris Lemson, Joost G. Daams, Reinout A. Bem, Job B. van Woensel

**Affiliations:** ^1^Pediatric Intensive Care Unit, Academic Medical Center, Emma Children’s Hospital, Amsterdam, Netherlands; ^2^Pediatric Intensive Care Unit, Radboud University Medical Center, Nijmegen, Netherlands; ^3^Medical Library, Academic Medical Center, University of Amsterdam, Amsterdam, Netherlands

**Keywords:** PARDS, fluid balance, management, critical care, children, lung edema

## Abstract

The administration of an appropriate volume of intravenous fluids, while avoiding fluid overload, is a major challenge in the pediatric intensive care unit. Despite our efforts, fluid overload is a very common clinical observation in critically ill children, in particular in those with pediatric acute respiratory distress syndrome (PARDS). Patients with ARDS have widespread damage of the alveolar–capillary barrier, potentially making them vulnerable to fluid overload with the development of pulmonary edema leading to prolonged course of disease. Indeed, studies in adults with ARDS have shown that an increased cumulative fluid balance is associated with adverse outcome. However, age-related differences in the development and consequences of fluid overload in ARDS may exist due to disparities in immunologic response and body water distribution. This systematic review summarizes the current literature on fluid imbalance and management in PARDS, with special emphasis on potential differences with adult patients. It discusses the adverse effects associated with fluid overload and the corresponding possible pathophysiological mechanisms of its development. Our intent is to provide an incentive to develop age-specific fluid management protocols to improve PARDS outcomes.

## Introduction

Pediatric acute respiratory distress syndrome (PARDS) is one of the most challenging disease entities in the pediatric intensive care unit (PICU). PARDS was recently defined by the Pediatric Acute Lung Injury Consensus Conference (PALICC) group as acute-onset hypoxic respiratory failure with new infiltrate(s) on chest radiography not fully explained by cardiac failure or fluid overload (1). This definition is based on the Berlin ARDS definition (2), with several adaptations to more adequately adhere to ARDS specifically occurring in children. Importantly, this includes the use of pulse-oximetry derived data to obtain the SpO_2_/FiO_2_ (S/F) ratio and oxygen saturation index (OSI) in the many patients in whom PaO_2_ measurements are unavailable. Of note, due to the relative novelty of the PALICC definition, the articles included in this review still employ the Berlin or older AECC definition of ARDS.

Patients with PARDS form a heterogeneous population within the PICU. This is due to the large age distribution, as PARDS affects infants to adolescents, and the variety of underlying triggers, such as pneumonia, sepsis, trauma, and aspiration. Importantly, differences in the pathophysiology between PARDS and adult ARDS are likely. This is best illustrated by the lower incidence (2.0–12.8/100,000 person-years versus 17.9–81.0/100,000 person-years) and lower mortality (18–35% versus 27–45%) of ARDS in children as compared with adults (1). Although there are potential age-related differences in lung immune and injury responses (3–5), the precise similarities and disparities in the pathophysiological mechanisms between PARDS and adult ARDS are not fully known. At the same time, the development of treatment strategies for PARDS leans heavily on adult ARDS studies and Bayesian approaches (6), as to circumvent lack of power in pediatric studies. It is needless to say that treating children with modalities tested in adults, while the current literature suggests important differences may exist between PARDS and adult ARDS, is highly undesirable. Further exploration of the specific age-related characteristics in relation to outcomes and treatments of PARDS is therefore highly needed.

Appropriate fluid management has become an important non-pulmonary treatment in the current “best practice” care of adult patients with ARDS. To prevent aggravation of lung edema, it is now generally recommended to avoid positive cumulative fluid balance and treat fluid overload after initial hemodynamic stabilization. Several studies in adult ARDS patients have studied the effect of a restrictive fluid management protocol (7). Most importantly, in the NHLBI Fluid and Catheter Treatment Trial (FACTT), it was demonstrated that an early restrictive fluid management protocol in adults with ARDS was effective in preventing fluid overload, which was associated with improved oxygenation and shorter duration of mechanical ventilation and ICU stay (8). This appears in line with the current adult literature on sepsis, which suggests that although early goal-directed therapy including (aggressive) fluid resuscitation is generally beneficial, it does not always lead to reduced mortality (9–11), and may even be associated with longer hospitalization and higher organ failure scores (11). Hence early fluid resuscitation is most likely beneficial, whereas early fluid overload is not. Several studies in a variety of populations of critically ill children, including those in the general PICU, post heart surgery, and those receiving renal replacement therapy, have also shown a positive association between early fluid overload and adverse outcome (12–17).

In this manuscript, we review the literature on the adverse effects of fluid overload with emphasis on potential physiological differences in fluid homeostasis between children and adults. In addition, we summarize the current evidence for a restrictive fluid management protocol specifically in PARDS by a systematic literature review.

## Age and Fluid Overload

In adults, multiple studies in patients with ARDS have indicated higher cumulative fluid balance is associated with worse disease outcome. There is an evident inverse correlation between number of ventilator-free days (VFDs) and cumulative fluid balance (18–23). In two randomized controlled trials comparing conservative and liberal fluid treatment, patients in the conservative fluid treatment arm were shown to have higher number of VFDs (8, 18). The correlation with mortality seems more ambiguous. Mortality has been shown to correlate with increasing cumulative fluid balance (21–23), but controversially not to a restrictive fluid treatment (8, 18). In one of the above mentioned trials, lung injury scores and oxygenation indices were also assessed, which both were significantly better when patients received a more restrictive fluid treatment. Consequently, these patients spent an average of 2.5 days less on ventilation as compared with patients treated in the liberal arm (8).

As mentioned above, there are distinct differences in incidence and outcome between adult ARDS and PARDS. As fluid overload is an important and much prevalent aspect of this disease entity, we deem it likely that physiological differences between adults and children in fluid homeostasis influence the progression and outcome of PARDS. Differences in fluid homeostasis exist between children and adults. In humans, aging is associated with a decrease in total body water (TBW) (24). The TBW exists of extracellular (ECW) and intracellular water (ICW). An often used measure to describe fluid compartments is the ECW/TBW ratio. With aging, the TBW declines from approximately 70% of body weight in infants to 60% in elderly (25). The ECW/TBW ratio initially decreases in childhood, but increases again after adulthood due to a relative increase of the ECW. While the lower ECW/TBW ratio in (young) children, together with a high metabolic rate and insensible loss, is a risk for dehydration, vice versa elderly with a higher ECW/TBW ratio may be more prone for fluid overload. For example, in elderly patients suffering from congestive heart failure, fluid overload is predominantly reflected by an increase in ECW (26). In addition, compensation mechanisms by means of redistribution of water between intracellular and extracellular compartments during fluid loss or overload may vary between children, adults, and elderly (27).

In critically ill children, including those with PARDS, it is likely, although yet hypothetical, that age-dependent changes in ICW and ECW as described above alter their susceptibility for adverse effects in end organs during fluid overload. For example, young children have a large brain to intracranial volume ratio as compared with adults, possibly making them relatively vulnerable to encephalopathy as a result of brain edema by excess of water. This is for example illustrated by the higher susceptibility for cerebral edema accompanying diabetic ketoacidosis in children when compared with adults (28, 29). In the lungs, extravascular water content normalized for body weight, and thus TBW, is higher in children as compared with adults, although it is age-independent when indexed for height (30). One could hypothesize that the relative large lung mass to body weight renders children less prone for development of lung edema by increased hydrostatic pressure during fluid overload. In addition, the rate of alveolar fluid clearance (AFC), which is important for the resolution of lung edema, may differ between children and adults. For example, regulation of expression of the epithelial sodium channel (ENaC), one of the essential epithelial channels for fluid clearance, appears age-dependent in the lungs and kidneys (31–33). ENaC is known to increase shortly before birth, and is implicated to decrease with aging. This indicates that children might have higher expression of ENaC than adults, possibly making them less vulnerable to develop lung edema. Similar age-dependent effects of keratinocyte growth factor on AFC have been suggested (34). Indeed, studies in animal models have indicated juveniles to be less prone to develop lung edema compared with their adult or senescent equivalents (4). Unfortunately, the relative paucity of human and animal studies comparing data from the whole age-spectrum with regard to mechanisms and effects of fluid overload at this point makes it undesirable to elaborate beyond speculation.

## Adverse Effects of Fluid Overload

Fluid overload in children with PARDS, and more broadly in all critically ill patients, may have an adverse effect on clinical outcome by leading to interstitial edema resulting in impaired oxygenation and perfusion of several tissues in the human body. Increased hydrostatic pressure as a consequence of intravascular fluid challenge is a major mechanism of this increase in extravascular water content. However, the development of interstitial edema by fluid overload is not solely due to heightened hydrostatic pressure as explained by the Starling forces (35). In addition, it may be explained by the proinflammatory response of the endothelium to increasing intravascular pressure with mechanical stress, which by itself adds to further leakage of proteins and fluid (35–37). Lately, interest is given to a specific part of the endothelium; the glycocalyx. The glycocalyx is a layer lining the luminal side of the endothelium, which consists of a complex web of membrane-bound proteoglycans, glycoproteins, and glycosaminoglycans (GAGs) (38). Degradation of the glycocalyx with shedding of components such as GAGs into the circulation leads to activation of proinflammatory and coagulation pathways resulting in increased endothelial permeability (39, 40). Interestingly, recent studies suggest that intravascular fluid overload may also cause glycocalyx degradation; however, the extent and clinical importance of this effect is yet unclear (41, 42).

Fluid overload can have deleterious consequences for many end organs. In patients with PARDS, the lungs appear particularly affected and the aggravation of lung edema is therefore considered a key mechanism of adverse outcome by fluid overload. Extravascular accumulation of protein-rich edema in the lungs is a hallmark of PARDS and occurs by disruption of the alveolar–capillary barrier. The principal proinflammatory, cell death, and proteolytic and coagulation pathways underlying this increased lung permeability have been reviewed elsewhere (43, 44). As a consequence of this increased alveolar–capillary permeability, patients with PARDS are likely to develop lung edema at a lower hydrostatic pressure threshold as is suggested by early studies in dogs (45, 46). In addition, increased systemic venous pressure as a consequence of fluid loading diminishes the lymphatic drainage, which also decreases the absorption rate of lung edema (47). Importantly, in children with acute respiratory failure, including PARDS, the extravascular lung water content, as measured by hemodynamic volumetric monitoring, correlates with both the extent of fluid overload and lung permeability, and is predictive of survival and duration of mechanical ventilation (48).

Other tissues, especially encapsulated organs such as the kidneys and liver, may be specifically prone for adverse effects of fluid overload by lower perfusion pressures. This could be explained by the capsule acting as an unyielding shield leading to a type of compartment syndrome (49–51). For example, a number of studies have shown that a high cumulative fluid balance is associated with the development of acute kidney injury (AKI) in critically ill patients (52–56). In particular, in the setting of ongoing oliguria despite hemodynamic stabilization, fluid overload may be harmful (49). Moreover, the healing of skin and other tissues, as well as the incidence of gastro-intestinal complications such as ileus, after surgery are negatively affected by interstitial edema resulting from fluid overload (57, 58).

## Effect of Fluid Overload in Pards

**Figure 1:**
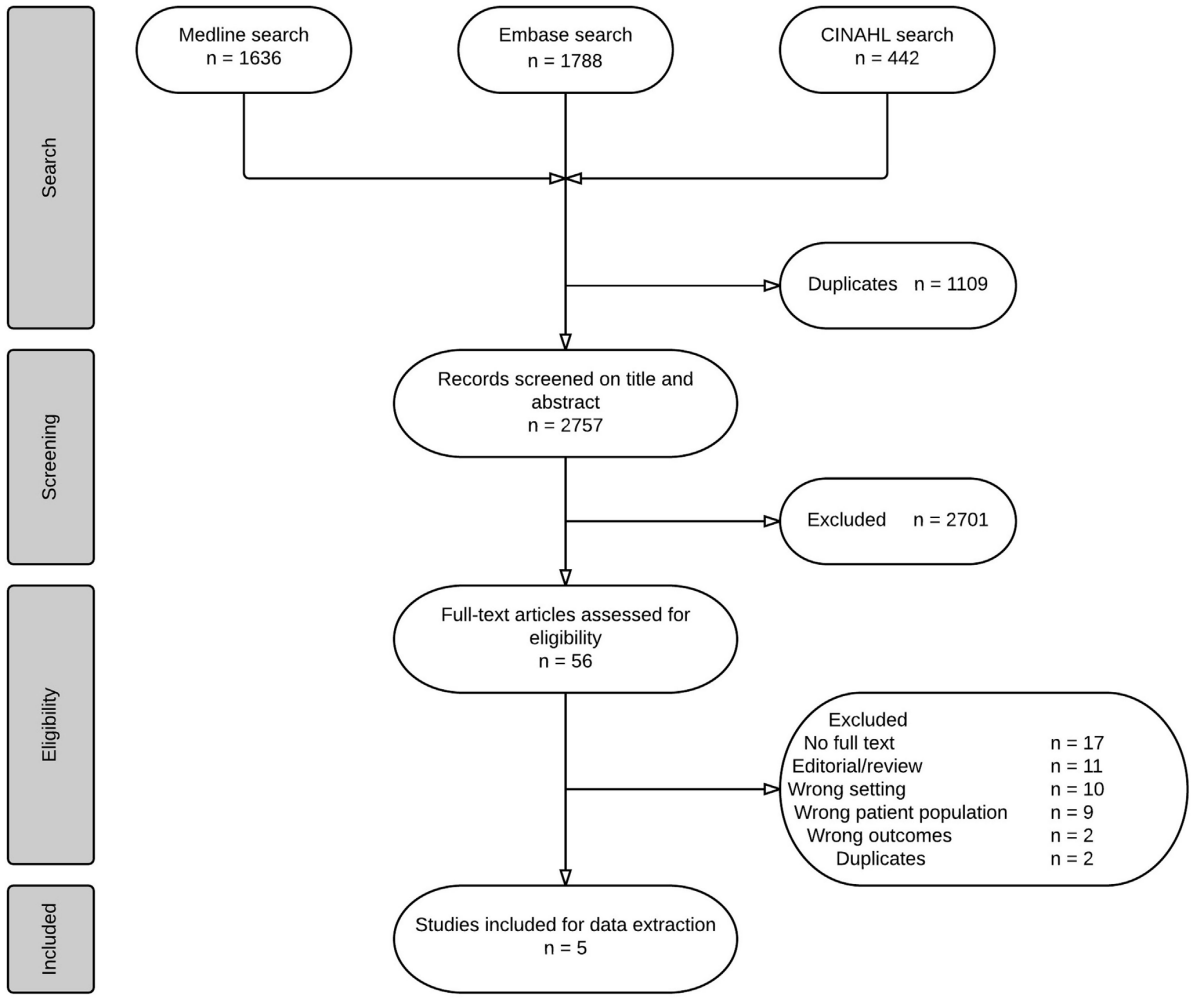
**Flow chart of selection process**.

In summary, fluid overload may be associated with negative outcome in critically ill children including those with PARDS, although important differences between children and adults in susceptibility and pathophysiology may exist. In order to address the current evidence that advocates or would support a restrictive fluid management specifically in PARDS, we performed a systematic review as described below. An overview of the search, screening and selection process of the studies can be found in Figure 1. Due to heterogeneity of the data, a meta-analysis was not deemed possible. The systematic literature search and critical appraisal are presented in the supplemental material of this manuscript.

### Overview of Included Studies

All studies included in this systematic review were part of a prospective or retrospective observational cohort or *post hoc* analysis. Table 1 holds the descriptions of the included studies. The correlations found between fluid balance and outcomes were broadly similar across the included articles. Table 2 provides a summary of the overall findings.

**Table 1:** **Summary of included articles**.

Study	Design	Outcome measure	Variable measure	Subjects	Definition ARDS	Outcome	NOS total
Flori et al. (59)	*Post hoc* analysis of a prospective observational study	PICU mortality, VFDs	Cumulative fluid balance in 10 ml/kg/day increments	313 children with ALI	ALI according to AECC definition	OR 1.08 (*p* = 0.02) mortality with increasing cumulative FB, VFDs (*p* = 0.02)	8
Valentine et al. (60)	Multicenter, retrospective cohort study	VFDs	Cumulative fluid balance and fluid overload (%)	168 children meeting ALI criteria	ALI according to AECC definition	Increasing cumulative FB at day 2–4 associated with fewer VFDs (*p* = 0.01/*p* = 0.01/*p* = 0.05). Cumulative fluid balance was not associated with mortality (*p* = 0.11)	7
Willson et al. (61)	*Post hoc* analysis of pediatric arm of prospective, randomized, placebo-controlled trial	In-hospital mortality, duration of MV, PICU, and hospital LoS, VFDs, OSI	Cumulative fluid balance	110 children with direct ALI/ARDS	ALI/ARDS according to AECC definition	Cumulative FB was associated with mortality (*p* < 0.001), VFDs (*p* < 0.001), PICU-free days (*p* = 0.14), and hospital-free days (*p* = 0.06). OSI increase of 0.52/l/m^2^ increase FB (*p* = 0.011)	8
Hu et al. (62)	Prospective study in 26 PICUs	Incidence, mortality and burden of AHRF and ARDS	Daily fluid balance	461 patients with AHRF, of which 306 (66%) ARDS	AHRF: PaO_2_ ≤50 mmHg or PaO_2_/FiO_2_ ≤250 mmHg for ≥6 h, needing FiO_2_ >30% and PEEP >2 cm H_2_O to maintain PaO_2_ >60 mmHg or SpO_2_ >90%. ALI/ARDS according to AECC definition	In AHRF: non-survivors had higher median FB (*p* = 0.079). Mortality of FB ≤10 ml/kg/day lower than >10 (*p* = 0.049). No specific ARDS data	6
Randolph et al. (63)	Prospective clinical trial	Extubation success (use of ERT) and duration of weaning	Cumulative fluid balance at extubation and start weaning	301 children with mechanical ventilation >24 h	No mention of ARDS definition	No relation cumulative FB with successful extubation. Duration of weaning with cumulative FB at ERT (HR 0.94, *p* = 0.06) and at extubation (HR 0.94, *p* = 0.051). No ARDS data only	7

*PICU, pediatric intensive care unit; ALI, acute lung injury; AECC, American-European consensus conference; VFDs, ventilator-free days; MV, mechanical ventilation; LoS, length of stay; OSI, oxygenation saturation index; FB, fluid balance; AHRF, acute hypoxemic respiratory failure; ERT, extubation readiness test*.

**Table 2:** **Description of findings on correlation between cumulative fluid balance and clinical outcomes**.

Outcome	Results	
Mortality	Significant association in three out of four studies in which mortality was assessed	
	Flori	*p* = 0.02
	Willson	*p* < 0.001
	Hu	*p* = 0.079
	Stratified in ≤10 or >10 ml/kg/day	*p* = 0.049
	Valentine	*p* = 0.11
VFDs	Significant association in three out of three studies in which VFDs were assessed	
	Flori	*p* = 0.02
	Valentine	*p* = 0.01
	Willson	*p* < 0.001
Oxygenation failure	Significant association in 1 out of 1 study in which degree of oxygenation failure was assessed	
	Willson	*p* = 0.011
Other	Willson	
	PICU-free days	*p* = 0.14
	Hospital-free days	*p* = 0.06
	Randolph	
	CFB at extubation associated with duration of weaning	*p* = 0.051

Mortality was one of the primary outcomes in this systematic review and was reported in most of the included studies. Flori et al. showed that mortality was associated with increasing cumulative fluid balance (grouped in sets of 10 ml/kg/day) in both bivariate and multivariate regression (OR 1.12, 95% CI 1.06–1.20, *p* < 0.001 and 1.08, 95% CI 1.01–1.15, *p* = 0.02, respectively) (59). Valentine et al. examined the same association in critically ill children with acute lung injury (ALI) according to the AECC definition of ALI (60). A trend of difference in cumulative fluid balance between survivors and non-survivors was found (*p* = 0.11). In the *post hoc* analysis by Willson et al. the increased cumulative fluid balance (as averaged per meter squared of body surface) was associated with in-hospital mortality on days 1–7 (*p* < 0.001) in children with ALI/ARDS (61). Hu et al. examined children with acute hypoxemic respiratory failure (AHRF) in China (62). A subgroup of these patients (*n* = 306, 66%) was identified according to the AECC criteria of ARDS. With regard to fluid data, they showed that non-survivors of AHRF had higher median fluid balances, although this was statistically not significant (*p* = 0.079). The mortality of patients with a daily fluid balance ≤10 ml/kg/day was lower than the group with a balance of >10 ml/kg/day (*p* = 0.049). There were no fluid data presented of only the ARDS subgroup. The last included study by Randolph et al. on the relation between weaning duration and fluid balance in mechanically ventilated children in the PICU held no data on mortality (63).

The other primary and important outcome measure was the number of VFDs measured at day 28 after admission or duration of ventilation. VFDs were used in three of the five included studies (59–61) and in all three fewer VFDs were associated with higher cumulative fluid balance. Valentine et al. reported that in days 2–4 of admission in the PICU, a higher cumulative fluid balance was associated with fewer VFDs (*p* = 0.01, 0.01, and 0.05 for days 2, 3, and 4, respectively) (60). Likewise, Willson et al. found the number of VFDs to be significantly inversely related to fluid balance (*p* < 0.001) (61). The study of Randolph et al. looked at fluid balance during weaning of ventilation and at the time of extubation (63). Their primary outcome was extubation success and secondary outcome was duration of weaning. Fluid balance was assessed at the time when an extubation readiness test (ERT) was performed or at the time of extubation, thus late in the course of disease. Cumulative fluid balance was neither at time of ERT nor at time of extubation associated with successful extubation (*p* = 0.56 and *p* = 0.77, respectively). Duration of weaning showed a non-significant relation with higher cumulative fluid balances at ERT (Hazard ratio 0.94, 95% CI 0.87–1.00, *p* = 0.06); however, it was (marginally) significant when predicted by the cumulative fluid balance values at extubation (Hazard ratio 0.94, 95% CI 0.87–1.00, *p* = 0.051). An important note to be taken into account when comparing the study of Randolph et al. to the others is that ARDS was not defined explicitly or analyzed separately.

One study found that a higher cumulative fluid balance showed a trend of an association with longer duration of stay in the PICU and hospital (61). This outcome was not addressed in other studies. These authors also compared the oxygenation saturation index (OSI) and fluid balance and found that for every liter per meter square body surface increase in fluid balance, the OSI increased with 0.52 point (*p* = 0.011) (61).

## Clinical Implications

Fluid overload early in PARDS progression seems to have a major impact on recovery. In particular *early* fluid overload is of main interest, as this seems to negatively affect clinical outcome. Late fluid overload may have a more modest effect on outcome (63), potentially because the cumulative fluid balance curve flattens out after the first few days of admission. Children with other causes of critical illness, such as shock or post-cardiac surgery, have shown the same trend of effect (56, 64). Likewise, in the general PICU population, early fluid overload is associated with longer duration of mechanical ventilation and worse oxygenation (13). The peak of illness, which is usually at or a few days after admission, coincides with the presence of early fluid overload. During this period of critical illness, one could speculate that children are especially vulnerable to the effects of fluid overload. Even more so, the positive fluid balance might enhance inflammatory processes (65), which may contribute to the association with adverse outcomes seen mainly in this first period. Vice versa, inflammation in itself could also worsen fluid accumulation by causing (endothelial) tissue damage (e.g., to the glycocalyx) and increasing permeability in the lungs. For pediatric critical care specialists, it is important to realize that children with PARDS appear particularly vulnerable for developing fluid overload already in the early phase of admission (15, 60). In order to prevent and/or adequately handle early fluid overload in daily practice, it is important to be very aware of the significance of this phenomenon. In particular, fluid treatment should probably be tailored more to the actual needs of patients, taking into account fluid responsiveness not only during resuscitation but also with regard to maintenance fluid therapy during the recovery phase (66).

Even though prevention is always more desirable than treatment, fluid overload is a common problem and needs to be treated. Fluid overload could be partially caused by an excess of fluid treatment or nutrients, which is an important pointer to base new fluid protocols on. To treat fluid overload, critically ill patients, both children and adults, often receive diuretics. In the population of critically ill children, furosemide has been shown to effectively achieve diuresis (67). In the particular setting of hypoproteinemic adult patients with ALI, treatment with the combination of furosemide and albumin improved oxygenation, hemodynamics, and fluid balance (19, 20). In animal models of ALI, furosemide also improved lung injury scores and oxygenation (68). The downside to diuretics is that patients are prone to become drug resistant, in which case increased amounts of the drug, a change of the administration route, or more drugs are needed. Moreover, loop diuretics such as furosemide are not compatible with all drugs, which must be taken into account when prescribing them (69).

Another more invasive method of reducing fluid overload is continuous renal replacement therapy (CRRT) (70). In multiple distinct populations of critically ill children, greater fluid overload at start of CRRT has been associated with mortality, even after adjusting for disease severity (14, 71–74). It is suggested that an earlier start of CRRT may result in better outcome as survivors have significantly less days in the PICU prior to start of CRRT than non-survivors do (71, 74). Sutherland et al. discuss the possibility of a threshold for CRRT initiation. For example, based on the recommendation by the American College of Critical Care Medicine, a threshold of >10% fluid overload is suggested for “an intervention” (14, 75). It seems that the choice of using CRRT to treat fluid overload can be defended; however, the optimal timing for initiation still remains to be elucidated and potential complications should be taken into account.

Although fluid restriction could be one of the strategies to prevent or overcome fluid overload, it is at the same time one of the main impeding factors in achieving energy requirements (76). Accomplishing sufficient nutrition is particularly important to prevent nutritional depletion, muscle wasting, and decreased immune function (70, 76) and is associated with better outcome (77). The development of clear fluid and nutritional protocols will be greatly beneficial in achieving optimal balance in both strategies. A Bayesian statistical approach could be the key to designing a randomized controlled trial in children with enough power to adequately assess the optimal fluid (and nutritional) protocol in children with PARDS. The gross effect of fluid overload seems similar in children and adults; however, the *size* of the effect cannot be readily compared due to the nature of the retrospective studies in children and lack of prospective randomized trials.

## Conclusion

Pediatric acute respiratory distress syndrome is a complex disease entity in need of multimodal therapy, of which fluid treatment is a major component. The current literature on fluid balance in PARDS is sparse but demonstrates that fluid overload is associated with worsening clinical outcome, such as fewer VFDs and worse oxygenation. Varying evidence was found on the relation with mortality and increasing fluid balance. In particular, *early* fluid overload was found to be of main influence. However, definite evidence by randomizing liberal versus restrictive fluid treatment in PARDS is lacking. This systematic review indicates the overall effect of fluid overload on outcomes is rather similar to the effect seen in adults; however, the size of effect cannot be compared at this time. Given the accumulating evidence for differences in (patho)physiology and outcome between children with PARDS and adults with ARDS, as well as known differences in fluid homeostasis related to aging, further study of the effects of specific tailor made fluid maintenance strategies in PARDS is highly warranted. In particular, the place for early diuretic and renal replacement therapy in order to limit fluid overload need to be explored. Further studies are needed to determine whether development of specific age-related fluid management protocols, taking into account all aspects of PARDS, is essential for optimal treatment of this patient group.

## Author Contributions

SI designed the review, conceptualized the systematic literature search, conducted the eligibility screening, assessed the quality of selected studies, extracted data from the selected studies, and drafted the initial manuscript. RA conceptualized the design of the review and critically revised the manuscript. JD conceptualized and performed the systematic literature search and critically revised the manuscript. JL conceptualized the design of the review and critically revised the manuscript. RB designed the review, conducted the eligibility screening, assessed the quality of selected studies, supervised the data extraction, collaborated in drafting the initial manuscript and critically revised the manuscript. JW designed the review, supervised the literature search and data collection, and critically revised the manuscript.

## Conflict of Interest Statement

The authors declare that the research was conducted in the absence of any commercial or financial relationships that could be construed as a potential conflict of interest.
