# Supplementary material for: Pediatric Acute Respiratory Distress Syndrome: Fluid Management in the PICU
Source: Front Pediatr. 2016 Mar 21;4:21. doi: 10.3389/fped.2016.00021 (PMC4800174; doi:10.3389/fped.2016.00021)
Supplement: Supplementary file 1 [file data_sheet_1.doc]

Supplementary Material

# Pediatric acute respiratory distress syndrome: fluid management in the PICU

S.A. Ingelse*, R.M. Wösten-van Asperen, J. Lemson, J.G. Daams, R.A. Bem, J.B. van Woensel

*** Correspondence:** S.A. Ingelse: s.a.ingelse@amc.uva.nl

# Supplementary Data

## Methods

### Search strategy and selection criteria

A systematic literature search was performed in Medline, EMBASE and CINAHL (from inception to August 2015), which contained search terms for fluid balance, overload and management combined with variants of terms such as “children”, “(pediatric) intensive care” and “acute respiratory distress syndrome”. Search strategies were developed in collaboration with a medical information specialist (J.G.D.). The full search strategy is presented in the supplemental methods. The two authors S.A.I. and R.A.B. independently conducted the eligibility screening of the identified publications. Studies were included if they assessed the relation between fluid balance, fluid overload or different fluid management regimes to outcome (ventilator free days/duration of ventilation, hospital or PICU length of stay, oxygenation and ventilation parameters and/or mortality) in a pediatric study population. Only full texts were included, conference abstracts were excluded. Moreover, studies should be based on original data and therefore reviews were excluded. Selection for inclusion consisted of initial screening based on titles and abstracts. Following this, full-text versions of the studies were assessed for eligibility. Conflicts about inclusion were solved by discussion between S.A.I. and R.A.B. Lastly, the references of included articles were screened for additional publications.

### Quality assessment and data extraction

Quality of the included articles was assessed by S.A.I and R.A.B. independently, using the Newcastle-Ottawa quality assessment scale (NOS). The NOS includes 8 criteria within the categories: selection, comparability and outcome measure. Studies could receive up to a maximum of 8 stars, as one criterion was not applicable. Eight stars was defined as very good quality, 6-7 as good quality, 4-5 as satisfactory quality and 0-3 as unsatisfactory quality. Reviewers reached consensus by discussion of the conflicts.

One reviewer (S.A.I.) extracted the data from the eligible studies. The primary outcome measures were mortality and duration of mechanical ventilation correlated to cumulative fluid balance data. Secondly, PICU and hospital length of stay and oxygenation indices were extracted if available.

## Results

### Study selection

The initial search generated 3866 articles. After careful selection 5 studies were included for data extraction (Figure 1). Studies were excluded for the reasons presented in Figure 1.

# 1.2.2 Quality appraisal

Using the Newcastle-Ottawa assessment scale, quality of the studies was examined. Most of the studies were of considerable good quality (6-8 on the NOS scale). The selection criteria were adequately met in all studies. However, one of the criteria, selection of the non-exposed cohort, could not be met in any of them as none of the studies employed a non-exposed cohort due to their design. Therefore, none of the studies received the full 9 stars appraisal and 8 was the maximum score. With regard to the comparability criteria, all but one (59) of the studies controlled for potential confounders. The main variable that was controlled for was the PaO_2_/FiO_2_ ratio at baseline. Randolph et al. however only adjusted for a few confounders such as age, gender and PRISM III scores. All studies were based on cohorts of patients and outcome was assessed by means of record linkage. Aside from the study by Valentine et al. (60), all manuscripts mentioned the adequacy of follow-up of their cohort.

# 1.2.3 Quantative analysis

The studies mostly had different outcome measures, which made the results very heterogenic. Moreover, nearly all studies mentioned only p-values of the associations between variables. Taken together, this made pooling of the study results not possible and a meta-analysis was therefore not performed.
