# Supplementary material for: Pediatric Acute Respiratory Distress Syndrome: Fluid Management in the PICU
Source: Front Pediatr. 2016 Mar 21;4:21. doi: 10.3389/fped.2016.00021 (PMC4800174; doi:10.3389/fped.2016.00021)
Supplement: Supplementary file 2 [file Table_1.doc]

Supplementary Material

# Pediatric acute respiratory distress syndrome: fluid management in the PICU

S.A. Ingelse*, R.M. Wösten-van Asperen, J. Lemson, J.G. Daams, R.A. Bem, J.B. van Woensel

*** Correspondence:** S.A. Ingelse: s.a.ingelse@amc.uva.nl

# Supplementary Table

Table S1: Systematic literature search in Medline

| Ovid **MEDLINE(R)** In-Process & Other Non-Indexed Citations and Ovid MEDLINE(R) 1946 to Present | | |
| --- | --- | --- |
| **#** | **Searches** | **Results** |
| 1 | exp child/ or exp adolescent/ or minors/ or exp schools/ or exp puberty/ or infant/ or infant,newborn/ | 3064092 |
| 2 | (youngster or pubert* or pubescent or prepubescent or school or schools or schoolkid* or schoolchild* or highschool* or kid or kids or underage* or youth? or boy or boys or girl? or sibbling* or preschool* or child or children or schoolchild* or adolescents or adolescence or juvenile or minors or teen or teens or teenager* or p?ediatric? or infant? or infancy or newborn? or toddler?).ab,kf,ti. | 1761063 |
| 3 | (child or p?ediatric? or adolescents or adolescence or juvenile).jw. | 456158 |
| 4 | or/1-3 | 3577252 |
| 5 | intensive care/ or intensive care units/ or respiratory care units/ | 53552 |
| 6 | (intensive care or icu or respiratory care units or critical care).ab,kf,ti. | 125780 |
| 7 | or/5-6 | 140043 |
| 8 | 4 and 7 | 42727 |
| 9 | intensive care units, pediatric/ | 5193 |
| 10 | (picu or pediatric intensive care).ab,kf,ti. | 5724 |
| 11 | or/9-10 | 8110 |
| 12 | 8 or 11 | 44250 |
| 13 | exp Water-Electrolyte Balance/ or Fluid Therapy/ or Water-Electrolyte Imbalance/ or Extravascular Lung Water/ or pulmonary edema/ | 63004 |
| 14 | (((electrolyte balance or electrolyte imbalance) and water) or lung edema or pulmonary edema).mp. [mp=title, abstract, original title, name of substance word, subject heading word, keyword heading word, protocol supplementary concept word, rare disease supplementary concept word, unique identifier] | 52646 |
| 15 | ((fluid adj2 (balance or overload or management or accumulation or intake or administration or infus* or therapy)) or early fluid or cumulative fluid or fluid intake minus output or fimo or "fluid in fluid out" or "fluid i o").ab,kf,ti. | 19315 |
| 16 | or/13-15 | 83404 |
| 17 | 12 and 16 | 1000 |
| 18 | acute lung injury/ or respiratory distress syndrome, adult/ | 18900 |
| 19 | (ards or pards or acute lung injur* or respiratory failure or respiratory insufficien* or respiratory distress or respiratory morbidity).ab,kf,ti. | 68244 |
| 20 | or/18-19 [ards] | 73019 |
| 21 | 4 and 16 and 20 [children and fluids and ards] | 858 |
| 22 | 12 and 16 and 20 [picu and fluids and ards] | 165 |
| 23 | 17 or 21 or 22 | 1693 |
| 24 | animals/ not humans/ | 3998271 |
| 25 | 23 not 24 | 1659 |
| 26 | remove duplicates from 25 | 1636 |

Table 3: Systematic literature search in EMBASE

| **Embase** Classic+Embase 1947 to 2015 August 14 | | |
| --- | --- | --- |
| **#** | **Searches** | **Results** |
| 1 | exp child/ or adolescent/ or hospitalized adolescent/ or "minor (person)"/ or school/ or college/ or high school/ or kindergarten/ or middle school/ or nursery school/ or primary school/ or exp adolescence/ | 3194924 |
| 2 | (youngster or pubert* or pubescent or prepubescent or school or schools or schoolkid* or schoolchild* or highschool* or kid or kids or underage* or youth? or boy or boys or girl? or sibbling* or preschool* or child or children or schoolchild* or adolescents or adolescence or juvenile or minors or teen or teens or teenager* or p?ediatric? or infant? or infancy or newborn? or toddler?).ab,kw,ti. | 2316148 |
| 3 | (child or p?ediatric? or adolescents or adolescence or juvenile).jx. | 571743 |
| 4 | or/1-3 | 3914947 |
| 5 | *intensive care/ or *newborn intensive care/ or *patient monitoring/ or *pediatric advanced life support/ or *intensive care unit/ | 89043 |
| 6 | (intensive care or icu or respiratory care units or critical care).ab,kw,ti. | 190894 |
| 7 | or/5-6 | 221283 |
| 8 | 4 and 7 | 60700 |
| 9 | (picu or pediatric intensive care).ab,kw,ti. | 9773 |
| 10 | 8 or 9 | 61614 |
| 11 | *fluid balance/ or *body fluid/ or *extravascular fluid/ or *interstitial fluid/ or *lung extravascular fluid/ or *lung fluid/ or *hypervolemia/ or *lung edema/ or *fluid retention/ or *fluid therapy/ | 31772 |
| 12 | (((electrolyte balance or electrolyte imbalance) and water) or lung edema or pulmonary edema).ab,kw,ti. | 21501 |
| 13 | ((fluid adj2 (balance or overload or management or accumulation or intake or administration or infus* or therapy)) or early fluid or cumulative fluid or fluid intake minus output or fimo or "fluid in fluid out" or "fluid i o").ab,kw,ti. | 27663 |
| 14 | or/11-13 | 68254 |
| 15 | 10 and 14 | 1155 |
| 16 | respiratory distress syndrome/ or acute lung injury/ or adult respiratory distress syndrome/ or neonatal respiratory distress syndrome/ or respiratory failure/ or acute respiratory failure/ or lung insufficiency/ | 107744 |
| 17 | (ards or pards or acute lung injur* or respiratory failure or respiratory insufficien* or respiratory distress or respiratory morbidity).ab,kw,ti. | 99773 |
| 18 | or/16-17 [ards] | 145396 |
| 19 | 4 and 14 and 18 [children and fluids and ards] | 989 |
| 20 | 10 and 14 and 18 [picu and fluids and ards] | 225 |
| 21 | 15 or 19 or 20 | 1919 |
| 22 | (animal/ or animal experiment/ or animal model/ or nonhuman/ or rat/ or mouse/ or (rat or rats or mouse or mice).ti.) not human/ | 5710482 |
| 23 | 21 not 22 | 1826 |
| 24 | remove duplicates from 23 | 1788 |

Table 4: Systematic literature search in CINAHL

| **CINAHL** | | |
| --- | --- | --- |
| **#** | **Query** | **Results** |
| S23 | S17 OR S21 OR S22 | 442 |
| S22 | S12 AND S16 AND S20 | 44 |
| S21 | S4 AND S16 AND S20 | 121 |
| S20 | S18 OR S19 | 16,285 |
| S19 | SU ards or pards or acute lung injur* or respiratory failure or respiratory insufficien* or respiratory distress or respiratory morbidity OR AB ards or pards or acute lung injur* or respiratory failure or respiratory insufficien* or respiratory distress or respiratory morbidity OR TI ards or pards or acute lung injur* or respiratory failure or respiratory insufficien* or respiratory distress or respiratory morbidity | 16,285 |
| S18 | (MH "Respiratory Distress Syndrome, Acute") OR (MH "Acute Lung Injury") OR (MH "Respiratory Failure") | 9,784 |
| S17 | S12 AND S16 | 365 |
| S16 | S13 OR S14 OR S15 | 13,461 |
| S15 | SU ((fluid N2 (balance or overload or management or accumulation or intake or administration or infus* or therapy)) or early fluid or cumulative fluid or fluid intake minus output or fimo or "fluid in fluid out" or "fluid i o") OR AB ((fluid N2 (balance or overload or management or accumulation or intake or administration or infus* or therapy)) or early fluid or cumulative fluid or fluid intake minus output or fimo or "fluid in fluid out" or "fluid i o") OR TI ((fluid N2 (balance or overload or management or accumulation or intake or administration or infus* or therapy)) or early fluid or cumulative fluid or fluid intake minus output or fimo or "fluid in fluid out" or "fluid i o") | 10,339 |
| S14 | SU (((electrolyte balance or electrolyte imbalance) and water) or lung edema or pulmonary edema) OR AB (((electrolyte balance or electrolyte imbalance) and water) or lung edema or pulmonary edema) OR TI (((electrolyte balance or electrolyte imbalance) and water) or lung edema or pulmonary edema) | 2,979 |
| S13 | (MH "Fluid Therapy") OR (MH "Fluid Balance (Iowa NOC)") OR (MH "Fluid Management (Iowa NIC)") OR (MH "Fluid Monitoring (Iowa NIC)") OR (MH "Fluid-Electrolyte Balance") OR (MH "Fluids and Secretions") OR (MH "Interstitial Fluid") OR (MH "Pulmonary Edema") | 8,674 |
| S12 | S8 OR S11 | 23,614 |
| S11 | S9 OR S10 | 12,812 |
| S10 | SU picu or pediatric intensive care OR AB picu or pediatric intensive care OR TI picu or pediatric intensive care | 4,661 |
| S9 | (MH "Intensive Care Units, Pediatric+") | 11,757 |
| S8 | S4 AND S7 | 21,358 |
| S7 | S5 OR S6 | 84,813 |
| S6 | SU intensive care or icu or respiratory care units or critical care OR AB intensive care or icu or respiratory care units or critical care OR TI intensive care or icu or respiratory care units or critical care | 81,586 |
| S5 | (MH "Respiratory Care Units") OR (MH "Intensive Care Units+") or (MH "Ventilator Patients") or (MH "Pediatric Advanced Life Support") or (MH "Critical Care") | 49,946 |
| S4 | S1 OR S2 OR S3 | 761,911 |
| S3 | JN child or p?ediatric? or adolescents or adolescence or juvenile | 351,86 |
| S2 | SU youngster or pubert* or pubescent or prepubescent or school or schools or schoolkid* or schoolchild* or highschool* or kid or kids or underage* or youth? or boy or boys or girl? or sibbling* or preschool* or child or children or schoolchild* or adolescents or adolescence or juvenile or minors or teen or teens or teenager* or p?ediatric? or AB youngster or pubert* or pubescent or prepubescent or school or schools or schoolkid* or schoolchild* or highschool* or kid or kids or underage* or youth? or boy or boys or girl? or sibbling* or preschool* or child or children or schoolchild* or adolescents or adolescence or juvenile or minors or teen or teens or teenager* or p?ediatric? or TI youngster or pubert* or pubescent or prepubescent or school or schools or schoolkid* or schoolchild* or highschool* or kid or kids or underage* or youth? or boy or boys or girl? or sibbling* or preschool* or child or children or schoolchild* or adolescents or adolescence or juvenile or minors or teen or teens or teenager* or p?ediatric? | 687,921 |
| S1 | MH "Child+" or MH "Adolescence" or MH "Schools, Elementary" OR MH "Schools, Middle" OR MH "Schools, Secondary" OR MH "Schools+ or MH "Students, Elementary" OR MH "Students, High School" OR MH "Students, Middle School" or MH "Puberty+" | 640,12 |
|
|
