# Supplementary material for: Pediatric Acute Respiratory Distress Syndrome: Fluid Management in the PICU
Source: Front Pediatr. 2016 Mar 21;4:21. doi: 10.3389/fped.2016.00021 (PMC4800174; doi:10.3389/fped.2016.00021)
Supplement: Supplementary file 3 [file Table_2.doc]

Supplementary Material

# Pediatric acute respiratory distress syndrome: fluid management in the PICU

S.A. Ingelse*, R.M. Wösten-van Asperen, J. Lemson, J.G. Daams, R.A. Bem, J.B. van Woensel

*** Correspondence:** S.A. Ingelse: s.a.ingelse@amc.uva.nl

# Supplementary Tables

Table S2: Systematic literature search in EMBASE

| **Embase** Classic+Embase 1947 to 2015 August 14 | | |
| --- | --- | --- |
| **#** | **Searches** | **Results** |
| 1 | exp child/ or adolescent/ or hospitalized adolescent/ or "minor (person)"/ or school/ or college/ or high school/ or kindergarten/ or middle school/ or nursery school/ or primary school/ or exp adolescence/ | 3194924 |
| 2 | (youngster or pubert* or pubescent or prepubescent or school or schools or schoolkid* or schoolchild* or highschool* or kid or kids or underage* or youth? or boy or boys or girl? or sibbling* or preschool* or child or children or schoolchild* or adolescents or adolescence or juvenile or minors or teen or teens or teenager* or p?ediatric? or infant? or infancy or newborn? or toddler?).ab,kw,ti. | 2316148 |
| 3 | (child or p?ediatric? or adolescents or adolescence or juvenile).jx. | 571743 |
| 4 | or/1-3 | 3914947 |
| 5 | *intensive care/ or *newborn intensive care/ or *patient monitoring/ or *pediatric advanced life support/ or *intensive care unit/ | 89043 |
| 6 | (intensive care or icu or respiratory care units or critical care).ab,kw,ti. | 190894 |
| 7 | or/5-6 | 221283 |
| 8 | 4 and 7 | 60700 |
| 9 | (picu or pediatric intensive care).ab,kw,ti. | 9773 |
| 10 | 8 or 9 | 61614 |
| 11 | *fluid balance/ or *body fluid/ or *extravascular fluid/ or *interstitial fluid/ or *lung extravascular fluid/ or *lung fluid/ or *hypervolemia/ or *lung edema/ or *fluid retention/ or *fluid therapy/ | 31772 |
| 12 | (((electrolyte balance or electrolyte imbalance) and water) or lung edema or pulmonary edema).ab,kw,ti. | 21501 |
| 13 | ((fluid adj2 (balance or overload or management or accumulation or intake or administration or infus* or therapy)) or early fluid or cumulative fluid or fluid intake minus output or fimo or "fluid in fluid out" or "fluid i o").ab,kw,ti. | 27663 |
| 14 | or/11-13 | 68254 |
| 15 | 10 and 14 | 1155 |
| 16 | respiratory distress syndrome/ or acute lung injury/ or adult respiratory distress syndrome/ or neonatal respiratory distress syndrome/ or respiratory failure/ or acute respiratory failure/ or lung insufficiency/ | 107744 |
| 17 | (ards or pards or acute lung injur* or respiratory failure or respiratory insufficien* or respiratory distress or respiratory morbidity).ab,kw,ti. | 99773 |
| 18 | or/16-17 [ards] | 145396 |
| 19 | 4 and 14 and 18 [children and fluids and ards] | 989 |
| 20 | 10 and 14 and 18 [picu and fluids and ards] | 225 |
| 21 | 15 or 19 or 20 | 1919 |
| 22 | (animal/ or animal experiment/ or animal model/ or nonhuman/ or rat/ or mouse/ or (rat or rats or mouse or mice).ti.) not human/ | 5710482 |
| 23 | 21 not 22 | 1826 |
| 24 | remove duplicates from 23 | 1788 |
