# Supplementary material for: Pediatric Acute Respiratory Distress Syndrome: Fluid Management in the PICU
Source: Front Pediatr. 2016 Mar 21;4:21. doi: 10.3389/fped.2016.00021 (PMC4800174; doi:10.3389/fped.2016.00021)
Supplement: Supplementary file 4 [file Table_3.doc]

Supplementary Material

# Pediatric acute respiratory distress syndrome: fluid management in the PICU

S.A. Ingelse*, R.M. Wösten-van Asperen, J. Lemson, J.G. Daams, R.A. Bem, J.B. van Woensel

*** Correspondence:** S.A. Ingelse: s.a.ingelse@amc.uva.nl

# Supplementary Tables

Table S3: Systematic literature search in CINAHL

| **CINAHL** | | |
| --- | --- | --- |
| **#** | **Query** | **Results** |
| S23 | S17 OR S21 OR S22 | 442 |
| S22 | S12 AND S16 AND S20 | 44 |
| S21 | S4 AND S16 AND S20 | 121 |
| S20 | S18 OR S19 | 16,285 |
| S19 | SU ards or pards or acute lung injur* or respiratory failure or respiratory insufficien* or respiratory distress or respiratory morbidity OR AB ards or pards or acute lung injur* or respiratory failure or respiratory insufficien* or respiratory distress or respiratory morbidity OR TI ards or pards or acute lung injur* or respiratory failure or respiratory insufficien* or respiratory distress or respiratory morbidity | 16,285 |
| S18 | (MH "Respiratory Distress Syndrome, Acute") OR (MH "Acute Lung Injury") OR (MH "Respiratory Failure") | 9,784 |
| S17 | S12 AND S16 | 365 |
| S16 | S13 OR S14 OR S15 | 13,461 |
| S15 | SU ((fluid N2 (balance or overload or management or accumulation or intake or administration or infus* or therapy)) or early fluid or cumulative fluid or fluid intake minus output or fimo or "fluid in fluid out" or "fluid i o") OR AB ((fluid N2 (balance or overload or management or accumulation or intake or administration or infus* or therapy)) or early fluid or cumulative fluid or fluid intake minus output or fimo or "fluid in fluid out" or "fluid i o") OR TI ((fluid N2 (balance or overload or management or accumulation or intake or administration or infus* or therapy)) or early fluid or cumulative fluid or fluid intake minus output or fimo or "fluid in fluid out" or "fluid i o") | 10,339 |
| S14 | SU (((electrolyte balance or electrolyte imbalance) and water) or lung edema or pulmonary edema) OR AB (((electrolyte balance or electrolyte imbalance) and water) or lung edema or pulmonary edema) OR TI (((electrolyte balance or electrolyte imbalance) and water) or lung edema or pulmonary edema) | 2,979 |
| S13 | (MH "Fluid Therapy") OR (MH "Fluid Balance (Iowa NOC)") OR (MH "Fluid Management (Iowa NIC)") OR (MH "Fluid Monitoring (Iowa NIC)") OR (MH "Fluid-Electrolyte Balance") OR (MH "Fluids and Secretions") OR (MH "Interstitial Fluid") OR (MH "Pulmonary Edema") | 8,674 |
| S12 | S8 OR S11 | 23,614 |
| S11 | S9 OR S10 | 12,812 |
| S10 | SU picu or pediatric intensive care OR AB picu or pediatric intensive care OR TI picu or pediatric intensive care | 4,661 |
| S9 | (MH "Intensive Care Units, Pediatric+") | 11,757 |
| S8 | S4 AND S7 | 21,358 |
| S7 | S5 OR S6 | 84,813 |
| S6 | SU intensive care or icu or respiratory care units or critical care OR AB intensive care or icu or respiratory care units or critical care OR TI intensive care or icu or respiratory care units or critical care | 81,586 |
| S5 | (MH "Respiratory Care Units") OR (MH "Intensive Care Units+") or (MH "Ventilator Patients") or (MH "Pediatric Advanced Life Support") or (MH "Critical Care") | 49,946 |
| S4 | S1 OR S2 OR S3 | 761,911 |
| S3 | JN child or p?ediatric? or adolescents or adolescence or juvenile | 351,86 |
| S2 | SU youngster or pubert* or pubescent or prepubescent or school or schools or schoolkid* or schoolchild* or highschool* or kid or kids or underage* or youth? or boy or boys or girl? or sibbling* or preschool* or child or children or schoolchild* or adolescents or adolescence or juvenile or minors or teen or teens or teenager* or p?ediatric? or AB youngster or pubert* or pubescent or prepubescent or school or schools or schoolkid* or schoolchild* or highschool* or kid or kids or underage* or youth? or boy or boys or girl? or sibbling* or preschool* or child or children or schoolchild* or adolescents or adolescence or juvenile or minors or teen or teens or teenager* or p?ediatric? or TI youngster or pubert* or pubescent or prepubescent or school or schools or schoolkid* or schoolchild* or highschool* or kid or kids or underage* or youth? or boy or boys or girl? or sibbling* or preschool* or child or children or schoolchild* or adolescents or adolescence or juvenile or minors or teen or teens or teenager* or p?ediatric? | 687,921 |
| S1 | MH "Child+" or MH "Adolescence" or MH "Schools, Elementary" OR MH "Schools, Middle" OR MH "Schools, Secondary" OR MH "Schools+ or MH "Students, Elementary" OR MH "Students, High School" OR MH "Students, Middle School" or MH "Puberty+" | 640,12 |
|
|
